# Retraction: Clinical significance of SPOCK2 expression signature for high-grade serous ovarian cancer patients

**DOI:** 10.3389/fgene.2026.1781969

**Published:** 2026-01-14

**Authors:** Frontiers Editorial Office

The journal retracts the 28 September 2022 article cited above.

Following concerns regarding the originality of the article, an investigation was conducted in accordance with Frontiers’ policies. It was found that the complaint was valid and that the article should be retracted, because of an unacceptable level of similarity to a preprint article published on Research Square by different authors:

Hao, J., Cao, Y., Gu, X., Ma, M., Wang, W., Xue, Y., An, R. (2021). Upregulated SPOCK2 Predicts a Worse Overall Survival and Correlates with Abundance of Tumor Infiltrating Immune Cells for High-Grade Serous Ovarian Cancer. Research Square [Preprint]. Available at: https://doi.org/10.21203/rs.3.rs-885744/v1 (Accessed January 6, 2026).

This retraction was approved by the Chief Editors of Genetics and the Chief Executive Editor of Frontiers. The authors have not responded to correspondence regarding this retraction.

Frontiers would like to thank the concerned reader who contacted us regarding the published article.

